# The Role of NADPH Oxidases (NOXs) in Liver Fibrosis and the Activation of Myofibroblasts

**DOI:** 10.3389/fphys.2016.00017

**Published:** 2016-02-02

**Authors:** Shuang Liang, Tatiana Kisseleva, David A. Brenner

**Affiliations:** ^1^Department of Surgery, University of California, San DiegoLa Jolla, CA, USA; ^2^Department of Medicine, University of California, San DiegoLa Jolla, CA, USA

**Keywords:** NADPH oxidase (NOX), liver fibrosis, myofibroblasts, reactive oxygen species (ROS), hepatic stellate cells (HSCs), hepatocytes

## Abstract

Chronic liver injury, resulted from different etiologies (e.g., virus infection, alcohol abuse, nonalcoholic steatohepatitis (NASH) and cholestasis) can lead to liver fibrosis characterized by the excess accumulation of extracellular matrix (ECM) proteins (e.g., type I collagen). Hepatic myofibroblasts that are activated upon liver injury are the key producers of ECM proteins, contributing to both the initiation and progression of liver fibrosis. Hepatic stellate cells (HSCs) and to a lesser extent, portal fibroblast, are believed to be the precursor cells that give rise to hepatic myofibroblasts in response to liver injury. Although, much progress has been made toward dissecting the lineage origin of myofibroblasts, how these cells are activated and become functional producers of ECM proteins remains incompletely understood. Activation of myofibroblasts is a complex process that involves the interactions between parenchymal and non-parenchymal cells, which drives the phenotypic change of HSCs from a quiescent stage to a myofibroblastic and active phenotype. Accumulating evidence has suggested a critical role of NADPH oxidase (NOX), a multi-component complex that catalyzes reactions from molecular oxygen to reactive oxygen species (ROS), in the activation process of hepatic myofibroblasts. NOX isoforms, including NOX1, NOX2 and NOX4, and NOX-derived ROS, have all been implicated to regulate HSC activation and hepatocyte apoptosis, both of which are essential steps for initiating liver fibrosis. This review highlights the importance of NOX isoforms in hepatic myofibroblast activation and the progression of liver fibrosis, and also discusses the therapeutic potential of targeting NOXs for liver fibrosis and associated hepatic diseases.

## Introduction

The main causes of hepatic fibrosis are chronic hepatitis B and C infection, autoimmune and biliary diseases, alcoholic steatohepatitis (ASH) and, increasingly, nonalcoholic steatohepatitis (NASH) (Bataller and Brenner, 2005). Liver fibrosis results from a sustained wound healing process in response to chronic liver injuries, and is characterized by accumulation of excessive extracellular matrix (ECM) proteins. Prolonged and excessive buildup of ECM proteins leads to pronounced distortion of hepatic vascular architecture due to formation of the fibrous scar, which promotes subsequent hepatocyte regeneration and hepatic endothelial dysfunction (Friedman, 2000). These processes facilitate the transition from liver fibrosis to cirrhosis, which may ultimately progress to more serious complications, such as portal hypertension due to increased resistance to portal blood flow, spontaneous bacterial peritonitis, and hepatic encephalopathy. Liver fibrosis is reversible, whereas cirrhosis, the end-stage consequence of fibrosis, is often irreversible and results in liver failure or the development of hepatocellular carcinoma (HCC) and death unless liver transplantation is done (Tsochatzis et al., 2014). Thus, it is of utmost importance to investigate the molecular and cellular mechanisms involved in the fibrogenic processes in order to design novel therapeutic interventions for liver fibrosis.

The major source of excessive ECM and fibrogenic mediators, such as collagen, is myofibroblasts. Recent studies indicate that the origin of myofibroblasts is liver intrinsic, and activated hepatic stellate cells (HSCs) and portal fibroblasts are believed to be the main precursors that give rise to hepatic myofibroblasts (Brenner et al., 2012). Upon liver injury, HSCs and portal fibroblasts undergo dramatic phonotypical changes by acquiring profibrogenic properties. In the normal liver, quiescent HSCs positive for adipocytes markers (PPARγ, SREBP-1c, and leptin) are the major cell type responsible for vitamin A storage (Bataller and Brenner, 2005). Upon activation by fibrogenic cytokines such as TGF-β1, angiotensin II, and leptin, quiescent HSCs trans-differentiate into myofibroblasts, possessing the properties of contractile, proinflammatory, and profibrogenic (Friedman, 2000). Activated HSCs express myogenic markers, such as α smooth muscle actin, c-myc, and myocyte enhancer factor–2 (Bataller and Brenner, 2005).

Accumulating clinical and pre-clinical data suggest that chronic liver injury results in the generation of oxidative stress, which disrupts lipids, proteins and DNA, induces necrosis/apoptosis of hepatocytes and amplifies the inflammatory response. Moreover, reactive oxygen species (ROS) mediate the progression of hepatic fibrosis by stimulating the production of profibrogenic mediators from Kupffer cells and circulating inflammatory cells and by directly activating HSCs to induce their trans-differentiation into myofibroblasts (Sánchez-Valle et al., 2012). Emerging evidence indicate that the nicotinamide adenine dinucleotide phosphate (NADPH) oxidases (NOXs) are sources of ROS, which play crucial roles in the progression of hepatic fibrosis (Aoyama et al., 2012; Paik et al., 2014). Seven NOXs isoforms have been identified in mammals so far. The major NOX isoforms expressed in the liver are NOX1, NOX2, and NOX4. NOX2 was the first discovered NOX in phagocytes, which plays important role in inflammation and host immune defense. HSCs and hepatocytes express NOX1, NOX2, and NOX4. It becomes increasingly clear that NOX-dependent ROS production is not limited to phagocytes because NOX enzymes are widely expressed and active in many different cell types from varies of tissues and organs. This review will focus on summarizing the roles of NOX isoforms that are distinctly expressed in different cell types in the liver.

## NADPH oxidases

ROS are defined as oxygen radicals, including reactive molecules, such as peroxide, superoxide, hydroxide, and singlet oxygen. In physiological conditions, ROS are generated during normal oxygen metabolism and play important roles in maintaining cellular homeostasis by orchestrating host defense, cell growth and signaling. However, ROS can also rapidly accumulate in large quantities during oxidative stress when cells encounter either endogenous or exogenous challenges. This, if not properly controlled, might lead to adverse cellular events, including irreversible cellular damage and death which may ultimately results in tissue damage and organ dysfunction (Devasagayam et al., 2004). ROS mediated oxidative stress is strongly associated with varieties of human diseases, including Parkinson's (Smeyne and Smeyne, 2013), Alzheimer (Aliev et al., 2014), cardiovascular (Robert and Robert, 2014), immunological (De Deken et al., 2014), pulmonary (Wong et al., 2013), renal (Ozbek, 2012), as well as liver diseases (Jaeschke, 2011).

In chronic liver diseases, pathological insults, such as ischemia-reperfusion, cholestasis or drug toxicity, induce hepatocyte death, which activates immune cells and promotes HSC transdifferentiation into collagen-producing myofibroblasts, which ultimately drives the development of hepatic fibrosis and cirrhosis. ROS accumulation in hepatocytes can cause cell death, which release damage-associated molecular patterns (DAMPs) that stimulates liver resident Kupffer cells and newly recruited immune cells to produce profibrogenic mediators. ROS is vital for HSC activation, resulting in the initiation of fibrosis. In the liver, several cellular machineries can generate ROS, including the mitochondrial respiratory chain, cytochrome P450 (CYP) family members, peroxisomes, xanthine oxidase, and NADPH oxidases. NADPH oxidase that produces ROS was first discovered in phagocytes, referred as gp91^*phox*^ (also known as NOX2), and serves as an important inflammatory mediator against invading bacteria. Recently, other NOX2 like molecules have been identified in various tissues. Due to the sequential and functional similarities of these enzymes to NOX2, these enzymes, together with NOX2 are collectively referred to as the NOX family. The NOX family genes encode proteins responsible for a transmembrane electron transport chain containing a flavocytochrome b, which transfers electrons donated by NADPH across biological membranes to form superoxide (O${}_{2}^{-}$) and hydrogen peroxide (H_2_O_2_) from molecular oxygen (Cross and Segal, 2004). Seven NOX family members have been identified so far, including NOX1, NOX2 (formerly known as gp91^phox^), NOX3, NOX4, NOX5, and dual oxidase Duox proteins (DUOX1 and DUOX2).

The phagocytic NOX (NOX2) core enzyme comprises several different subunits that interact with each other to form an active enzyme complex, including NOX2 (gp91^*phox*^), p40^*phox*^ (*PHOX* for phagocyte oxidase), p47^*phox*^, p67^*phox*^, p22^*phox*^, Rac2, and Rap1A, which is responsible for superoxide production upon agonist stimulation. In the resting stage, two integral membrane proteins—gp91phox and p22phox, form a large heterodimeric subunit flavocytochrome b_558_ (cyt b_558_). Three of the regulatory proteins, p40^*phox*^, p47^*phox*^, and p67^*phox*^ form a complex in the cytosol (Groemping and Rittinger, 2005; Sumimoto et al., 2005). Upon stimulation (e.g., exposure of cells to microorganisms or inflammatory mediators), p40^*phox*^ is highly phosphorylated, resulting in the entire cytosolic complex translocation to plasma membrane and association with flavocytochrome b_558_. The whole NOX complex activation also requires the association of two low-molecular-weight guanine nucleotide-binding proteins, Rac2 GTPase and Rap1A (Diebold and Bokoch, 2001). Then the activated complex transfers electrons from the cytosolic NADPH to oxygen on the luminal or extracellular region (Koga et al., 1999). The expression of NOX2 is induced by interferon-γ (IFN-γ) through a transcription factor protein complex, called hematopoiesis-associated factor (HAF1), which is comprised of PU.1, interferon regulatory factor 1 (IRF-1), and interferon consensus sequence-binding protein (ICSBP) (Eklund et al., 1998).

NOX1 is identified as the first homolog of NOX2, and shares 60% amino-acid identity with NOX2 (Suh et al., 1999). NOX1 is widely expressed in many cell types, such as vascular smooth muscle cells (VSMCs), endothelial cells, astrocytes, and microglia. In liver, NOX1 is expressed in HSCs, ECs, and hepatocytes. However, the subcellular localization of NOX1 remains nebulous. It was suggested that NOX1 is a plasma membrane protein, and potentially resides in caveolin 1-containing lipid rafts (Hilenski et al., 2004; Zuo et al., 2005). Similar to NOX2, the activation of NOX1 also requires regulatory subunits, known as NOX organizer 1 (NOXO1) and NOX activator 1 (NOXA1), which are homologs of p47^*phox*^ and p67^*phox*^, respectively (Bánfi et al., 2003; Cheng and Lambeth, 2004). In addition, p22^*phox*^ and Rac GTPase are also required for NOX1 activation. Expression of NOX1 is also highly regulated. Its mRNA is induced by the growth factors including platelet-derived growth factor (PDGF), and angiotensin and phorbol esters (Suh et al., 1999; Lassègue et al., 2001).

NOX4, which is first discovered in kidney, shares 39% sequence homology with NOX2 (Geiszt et al., 2000). Its activity requires direct interaction with p22^*phox*^, but independent of the interaction with any cytosolic regulatory subunits (Ambasta et al., 2004). Moreover, Poldip2, a polymerase delta-interacting protein, has been shown to be associated with p22^*phox*^, which ultimately increases NOX4 enzymatic activity in VSMCs (Lyle et al., 2009). Similar to NOX1, NOX4 expression can also be regulated by angiotensin II. Moreover, TGFβ is also a potent regulator of NOX4 mRNA (Sturrock et al., 2006; Bondi et al., 2010).

## Origins and activation of hepatic myofibroblasts

Chronic liver injury of all etiologies can promote liver fibrosis, a wound healing process whose hallmark is the formation of fibrous scar constituted by ECM. The main producer of extracellular matrix proteins in the liver is myofibroblast, a terminally differentiated cell type that plays a critical role in wound healing and connective tissue remodeling. Not only possessing the ECM synthesizing features of fibroblasts, myofibroblast also has the contractile functions similar to the smooth muscle cells (Hinz et al., 2012). Under the self-limiting and homeostatic tissue repair processes, such as wound healing, myofibroblasts are induced and differentiated from their precursors, migrate to the site of injury, function to produce ECM proteins to contract the wound, and finally undergo apoptosis once injury is resolved. However, these processes can become uncontrolled when the myofibroblasts activities become excessive and persist due to the inability to undergo apoptosis, for example. This will lead to overwhelming ECM deposition, resulting in fibrosis and eventually cirrhosis (Watsky et al., 2010). In addition to the normal tissue repair and wound healing responses, myofibroblasts also contribute to regeneration, inflammation, angiogenesis, and stromal reaction during tumorigenesis. Although, myofibroblasts differ from fibroblasts by their ability of the former to de novo synthesize of α-smooth muscle actin (α-SMA), this is not an absolute requirement to define a cell as myofibroblast. Instead, the most reliable features of myofibroblasts are secretion of extracellular matrix, development of adhesion structures, and formation of contractile bundles (Hinz, 2010). Several novel markers of myofibroblasts, such as endosialin for tumor-associated myofibroblasts (Christian et al., 2008), P311 for hypertrophic scar myofibroblasts (Tan et al., 2010), and integrin α11β1 for human corneal myofibroblasts (Carracedo et al., 2010), have been recently identified. However, none of these markers are specific for myofibroblasts, and they play distinct roles in various types of fibroblasts, therefore affecting myofibroblasts differentiation in a tissue- and context- dependent manner. Nonetheless, reliable and unique markers for myofibroblasts remain to be defined.

Myofibroblasts are absent in healthy liver, but they are induced and activated from their precursor cells in response to hepatic injury. Although, the origin of myofibroblasts is yet unclear, three possible sources of myofibroblasts precursors in the liver have been proposed. The first possible source is the group of resident cells from the mesodermal origin that can potentially become myofibroblasts. This includes HSCs, portal fibroblasts, smooth muscle cells, and fibroblast around the central veins, which are different from hepatocytes, Kupffer cells, and sinusoidal endothelial cells. The second group of possible precursors of myofibroblasts are hepatocytes, cholangiocytes, and endothelial cells that can undergo epithelial or endothelial mesenchymal transition (EMT). However, several fate tracing and genetic labeling studies argued that hepatocytes or cholangiocytes did not undergo EMT in liver fibrosis models (Humphreys et al., 2010; Scholten et al., 2010; Taura et al., 2010). As for renal fibrosis, recent two studies argued that renal epithelial cells can undergo EMT, relaying signals to the interstitium to promote myofibroblast differentiation and fibrogenesis rather than directly giving rise to myofibroblasts population (Grande et al., 2015; Lovisa et al., 2015). Finally, bone marrow (BM)-derived cells consisting of fibrocytes and circulating mesenchymal cells can migrate into fibrotic liver tissue, transform into myofibroblasts and may contribute in the progression of liver fibrosis (Russo et al., 2006). Thus, these cells could also be possible precursors of myofibroblasts. However, a recent study using bone marrow (BM) chimeric mice reconstituted from transgenic collagen reporter mice suggested that BM cells had negligible contribution in collagen production during hepatic fibrosis (Higashiyama et al., 2009).

Among different mesenchymal cell types, the vitamin A-containing lipocytes (HSCs) capable of producing type III collagen was the first identified myofibroblast precursor in the liver (Kent et al., 1976). Since then, much focus has been put on HSCs to identify the origin of myofibroblasts. Upon liver injury, HSCs are activated, and converted from quiescent vitamin-A rich cells to proliferative, fibrogenic, and contractile myofibroblasts (Friedman, 2008). HSCs are regarded as the “warehouse” of retinoid droplets that exhibit blue-green autofluorescence when excited by UV light. However, cells that are absent of retinoid droplets are distinct from HSCs, which undergo a PDGF-mediated conversion into myofibroblasts (Kinnman et al., 2003). These cells are thought to be portal fibroblasts that are accumulated around bile ducts, and might play a critical role in the early stage of bile duct ligation (BDL) induced fibrosis (Tuchweber et al., 1996). Moreover, liver fibrosis seems to develop predominantly from the portal area and progress from there, irrespectively of the underlying etiology. Therefore the role of portal fibroblasts in the development of fibrosis may be more important than generally assumed.

## NOXs in HSC activation

**Figure 1:**
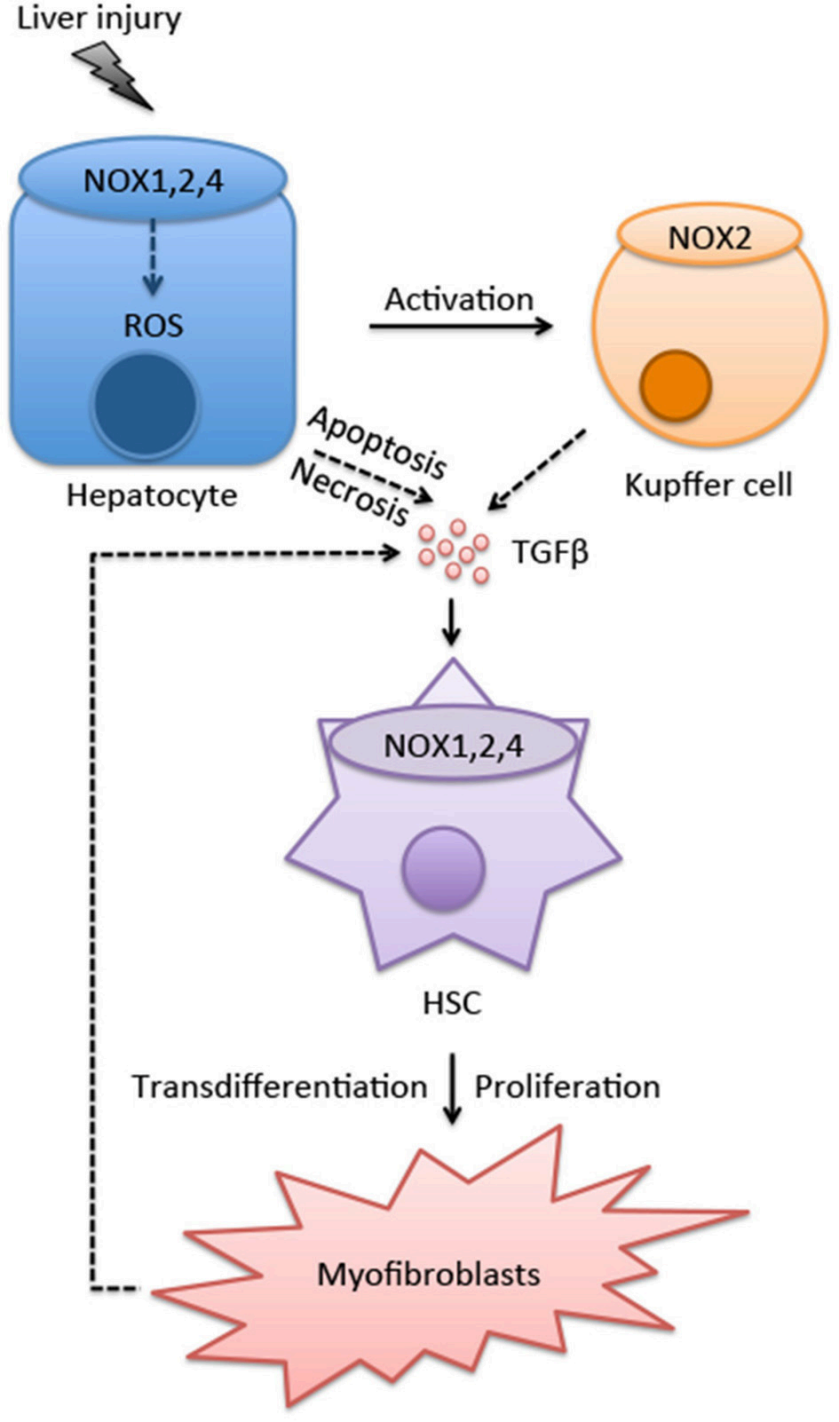
**The role of NOXs in myofibroblasts activation**. The interactions between hepatocytes, Kupffer cells and HSCs promotes myofibroblast activation. Various NOX isoforms expressed in different cell types in the liver play crucial roles during this process. After exposure to hepatic insults, such as ischaemia/reperfusion (IR) injuries, alcohol abuse, viral infection, hyper-nutrition, and cholestasis, ROS is produced through NOXs in hepatocytes. Increased oxidative stress also induces hepatocyte apoptosis/necrosis, resulting in release of DAMPs that activate Kupffer cells. Injured hepatocytes and activated Kupffer cells secrete proinflammatory and profibrogenic cytokine TGFβ, which promotes the differentiation of HSCs into myofibroblasts (see text for further details).

Upon liver injury, quiescent HSC become activated. The activation process is characterized by the loss of vitamin-A containing droplets, *de novo* synthesis of a-SMA, collagen and ECM proteins, and increased contractile and cell survival. The activation of HSC is a complex process, which involves the contribution of extracellular stimuli and different cell types, including parenchymal cells, immune cells. NOX proteins and NOX-derived ROS play a key role during HSC activation (Figure 1). ROS are produced in defined cellular compartments, but diffuse throughout the cell (e.g., superoxide) or across the plasma membranes (e.g., H_2_O_2_). ROS, when present at low levels, could serve as secondary messengers in response to a variety of cellular stimuli. For instance, it has been shown that low amount of hydrogen peroxide (H_2_O_2_) can act as second messenger that plays a critical role in the initiation and amplification of signaling during lymphocyte activation (Reth, 2002). In contrast, high level of ROS can be toxic and may lead to cell death. Although, low levels of ROS promote HSC to produce collagen and proliferate, while high-level toxic amount of ROS can induce death of HSCs (Novo et al., 2006).

NOXs are highly upregulated in patients with organ fibrosis, such as heart, lung, kidney, and pancreas. In consistence with their role in human fibrosis, NOX-derived ROS is also essential for multiple organ fibrosis in mice (Paik et al., 2006). In addition, during HSC activation, NOX mediates a number of fibrogenic responses induced by different agonists, including Ang II, PDGF, leptin, and TGFβ. Moreover, phagocytosis of apoptotic bodies by HSC leads to NOX activation and procollagen α1 (I) expression (Zhan et al., 2006). The expression of NOX isoforms is different among different types of liver resident cells. Kupffer cells only express phagocytic NOX2, whereas hepatocytes and HSC express various NOX isoforms, including phagocytic NOX2 and non-phagocytic NOX1, NOX4, DUOX1, and DUOX2. Endothelial cells mainly express NOX1, NOX2, and NOX4. Upon liver injury, NOX isoforms in HSC are strongly upregulated when quiescent HSC become activated myofibroblasts (Paik et al., 2011; Aoyama et al., 2012). HSCs can also fine-tune ROS production by expressing the regulatory subunits of NOX complexes, including p22^*phox*^, p40^*phox*^, p47^*phox*^, p67^*phox*^, NOXO1, NOXOA1, and Rac1. It has been shown that the p47phox regulatory subunit is induced in HSCs activated upon BDL-mediated fibrosis (De Minicis et al., 2010). At resting stage, human HSCs express low levels of both catalytic and regulatory NOX components, including NOX1, NOX2, and p47^*phox*^. However, these NOX subunits are highly upregulated in HSCs from patients with fibrotic liver diseases (Bataller et al., 2003). Moreover, NOX1 and NOX4 protein levels were increased in human livers with cirrhosis compared with normal controls (Lan et al., 2015).

## NOX4 in HSCs

A complex network involving paracrine/autocrine signals in parenchymal and nonparenchymal cells is required for HSCs activation and differentiation into myofibroblasts. Accumulating evidence has suggested that NOXs are the key mediators in HSC activation, which promotes hepatic fibrosis. TGFβ is the most potent regulator promoting collagen production and α-SMA expression in myofibroblasts from various organs, including liver (Gressner et al., 2002), kidney (Desmoulière et al., 2003), lung (Hardie et al., 2009), and heart (Kuwahara et al., 2002). During the initiation and progression of liver fibrosis, TGFβ plays a crucial role in regulating HSC activation, as well as inducing hepatocyte apoptosis, which leads to the secretion of cytokines, chemokines and microparticles that are critical for HSC and Kupffer cell activation. A number of studies have shown that NOX4 is essential for TGFβ-induced myofibroblast activation and fibrogenic responses such as collagen production in different organs, including lung (Hecker et al., 2009), kidney (Bondi et al., 2010), heart (Cucoranu et al., 2005; Chan et al., 2013), and prostate (Sampson et al., 2011). Moreover, although NOX4 can be induced by TGFβ in several different organs (Cucoranu et al., 2005; Sturrock et al., 2006; Bondi et al., 2010; Boudreau et al., 2012), the mechanism involved is controversial among different organs. In kidney and lung myofibroblasts, TGFβ induces NOX4 expression and ROS generation through the classical Smad2/3 pathway (Sturrock et al., 2006; Bondi et al., 2010), whereas NOX4 ugregulation is upstream of Smad2/3 activation in cardiac myofibroblasts (Cucoranu et al., 2005). In liver fibrosis, TGFβ induces NOX activity and ROS production during HSC activation, which plays key role in hepatic myofibroblasts activation (Proell et al., 2007). In BDL- or CCl4-mediated liver fibrosis, NOX4 expression and its activity are upregulated via a TGFβ-Smad3 dependent manner in HSCs (Jiang et al., 2012; Sancho et al., 2012). In addition, NOX4 expression correlates with the fibrotic scores in patients with hepatitis C virus infection or NASH (Sancho et al., 2012; Bettaieb et al., 2015). ROS production and the expression of fibrogenic markers are dramatically reduced in HSCs deficient in NOX4 (Jiang et al., 2012). Moreover, experiments using siRNA against NOX4 attenuated HSC activation, and more importantly, knocking down NOX4 in activated myofibroblasts could reverse the fibrotic phenotypes. Knocking down NOX4 in activated HSCs decreased the expression a-SMA and collagen production with no influence on TGFβ1 expression and phosphorylation of Smad2/3. These indicate that NOX4 activation and the following ROS production are downstream of TGFβ-Smad2/3 signaling pathway (Sancho et al., 2012).

Patients with hepatic fibrosis as a result of various chronic liver injuries, including viral infection, toxin, metabolic disorders, alcohol abuse, and cholestasis, have a breach in gut barrier function. This leads to compromised intestinal permeability that allows the entry of bacteria-derived components (e.g., LPS and CpG-containing DNAs through portal circulation and eventually into the liver, where they activates liver immune cells via acting on toll-like receptors (TLRs) (Seki and Brenner, 2008; Yang and Seki, 2012). TLRs are a group of pattern recognition receptors that recognize their cognate ligands with as either pathogen-associated molecular patterns (PAMPs) or DAMPs. Although, both murine and human HSCs express multiple TLRs (Wang et al., 2009), they respond poorly to TLR ligands, such as Gram-positive bacterial products peptidoglycan (PGN) and lipoteichoic acid (LTA) (Paik et al., 2006). Upon ligand engagement, TLRs are activated and transduce signals through downstream adaptor molecules MyD88 and TRIF to induce the expression of proinflammatory cytokines and chemokines. These inflammatory mediators then recruit KCs and circulating monocytes/macrophages, which produce TGFβ1 to drive the differentiation of HSC into myofibroblast (Seki and Brenner, 2008; Aoyama et al., 2010). Although, the LPS-TLR4 axis is crucial for hepatic fibrogenesis and liver fibrosis is dramatically attenuated in germ-free mice (Seki et al., 2007), the roles of NOX and ROS have not been extensively studied in the context of regulating LPS-TLR4 mediated inflammatory or fibrogenic responses in HSCs. In macrophages, NOX inhibitor DPI or siRNA against p22^phox^ significantly decreased LPS-TLR4-mediated activation of endoplasmic reticulum (ER)-stress sensor kinase IRE1α and its downstream target, the transcription factor XBP1 (Martinon et al., 2010). Moreover, it has been shown that the C-terminal domains of NOX4 and TLR4 directly interact with each other in HEK293T cells (Park et al., 2004). Consistently, in human aortic endothelial cell (HAECs), overexpression of the C-terminal region of NOX4 inhibited nuclear factor-kappaB (NF- κB) activation in response to LPS. NOX4 downregulation using siRNA resulted in reduced ROS production and less expression of adhesion molecule (ICAM-1) and chemokines such as CXCL8 and MCP-1 in response to LPS (Park et al., 2006). Therefore, NOX4 and NOX4-mediated ROS generation may regulate LPS induced NF-κB activation and its downstream signaling pathway in HSC activation and profibrogenic effects of myofibroblasts.

## NOX1/NOX2 in HSCs

In addition to the TGFβ-NOX4 axis-mediated activation of HSC and expression of fibrogenic factors, other NOX isoforms, including NOX1, NOX2, and NOX2 regulatory subunit p47^*phox*^, are also reported to orchestrate the progression of hepatic fibrosis (Aram et al., 2009; Jiang et al., 2010; Paik et al., 2011). p47^*phox*−∕−^ mouse was the first genetic model of NOX inhibition in the study of HSC function in liver fibrosis. After BDL-induced liver injury, p47^*phox*−∕−^ mice showed attenuated liver injury and fibrosis compared with WT mice. HSCs produce more type I collagen and TGFβ when treated with Angiotensin II (Ang II) (Yoshiji et al., 2001). Ang II also stimulates ROS production, and activates intracellular signaling pathways involving PKC, PI3K-Akt, MAPKs, ERK, and c-Jun, which presumably promotes HSC migration and proliferation. Consistent with this notion, HSC isolated from p47^*phox*−∕−^ mice had reduced cell motility and expansion capacity, and displayed a reduced fibrogenic response to Ang II (Bataller et al., 2003). Although the detailed molecular mechanism underlying Ang II-induced NOX activation and ROS production is still unclear, studies have indicated that Ang II induces ROS production through two consecutive events in vascular smooth muscle cells: the first event, which occurs within 30 s after Ang II stimulation, is dependent on PKC-mediated phosphorylation of p47^*phox*^. Phosphorylated p47^*phox*^ then translocates to the membrane where it binds to and facilitates the activation of NOX1 and/or NOX2. The second event that leads to sustained NOX activation and the following ROS production induced by Ang II (peaked at 30 min) requires the activation of Rac GTPase. Ang II-mediated Rac activation is PI3K, EGFR, and c-Src dependent (Seshiah et al., 2002). In order to keep the prolonged signal induced by Ang II, the expression levels of NADPH catalytic subunits, as well as the regulatory subunits p47phox and p22phox are also upregualted during Ang II stimulation (Fukui et al., 1997; Lassègue et al., 2001; Touyz et al., 2002). Similarly in HSCs, the mRNA levels of both NOX1 and NOX4 are increased upon Ang II treatment (Aoyama et al., 2012).

Proliferation of HSCs is a prerequisite that mediates the proper function of HSC-derived myofibroblasts and fibrogenic response in general. It has been suggested that NOX1 is crucial for promoting HSC proliferation and ROS production in bile duct ligated mouse liver. The underlying molecular mechanism is proposed to involve oxidation and inactivation of phosphatase and tensin homolog (PTEN), leading to the activation of AKT/FOXO4/p27(kip) signaling pathway that promotes HSC proliferation and fibrogenesis following BDL-induced liver injury (Cui et al., 2011). Platelet-derived growth factor (PDGF) is considered the most potent mitogen that promotes HSC proliferation (Pinzani et al., 1989). It has been shown that NOXs play a crucial role in this process (Adachi et al., 2005). PDGF induces HSC proliferation through ROS production, and NOX inhibitor (DPI) or p38 MAPK inhibitor suppressed PDGF-induced ROS production and HSC proliferation (Adachi et al., 2005). Similarly, PDGF stimulates NOX-dependent proliferation of activated pancreatic stellate cells (PaSCs) in chronic alcoholic pancreatitis/fibrosis (Hu et al., 2007). Mechanistically, PDGF stimulation promotes NOX1 expression and ROS production. In line with this, NOX1 is critical in PDGF stimulated vascular hypertrophy through activation of PKCδ (Fan et al., 2005) and inducing transcription factor (ATF)-1 (Katsuyama et al., 2005). Additionally, it has been shown that the transcription factor AP1 binding site is critical for the promoter activity of NOX1 (Cevik et al., 2008). Recently, it has also been shown NOX1/NOX4 inhibitor suppressed PDGF mediated ROS production and proliferative gene expression in primary mouse HSCs (Lan et al., 2015).

## NOXs in hepatocytes

Hepatocytes injury and death are important triggers of myofibroblasts activation. Dying hepatocyte can release DAMPs that induce the secretion of cytokines and chemokines from KCs/macrophage that eventually results in HSC activation and liver fibrosis. TGFβ1, secreted by active KCs/macrophage upon liver injury, can promote hepatocyte apoptosis (Oberhammer et al., 1992). The classical TGFβ mediated signaling pathway requires the binding of TGFβ1 to the TGFβ receptor (TGFβRI and II), leading to the phosphorylation and activation of Smad2/Smad3. Smad2/3 then interact with Smad4 to form an active Smad complex that enters the nucleus and binds to the promoter regions of TGFβ target genes to initiate their transcription. NOX4 is one of the TGFβ target genes. A key finding is that the expression of NOX4 is increased in NASH patients compared with healthy controls (Bettaieb et al., 2015). In hepatocytes, NOX4 expression is induced by TGFβ, and the activity of NOX4 is crucial for TGFβ mediated apoptosis of hepatocytes (Carmona-Cuenca et al., 2008). For instance, knocking down NOX4 in human hepatocytes cell lines (HepG2 and Hep3B) resulted in impaired NOX activity, caspase activation and cell death induced by TGFβ1 (Carmona-Cuenca et al., 2008). In rat fetal hepatocytes, TGFβ1 induces apoptosis through upregulating NOX4-mediated ROS production, followed by down-regulation of pro-survival protein Bcl-x_L_, which ultimately results in the loss of mitochondrial membrane potential and initiation of cytochrome C release (Herrera et al., 2001). Additionally, TGFβ1-induced NOX4 activity also increases the levels of pro-apoptotic proteins BIM and BMF (Ramjaun et al., 2007; Caja et al., 2009), and thus further amplifies apoptotic signals. NOX4-derived ROS regulates the transcription of Bcl-x_L_ and Bmf, whereas its regulation of BIM occurs post-transcriptionally (Caja et al., 2009). Moreover, EGF blocks TGFβ-induced NOX4 expression and hepatocytes death in a MEK/ERK and PI3K/Akt dependent manner (Carmona-Cuenca et al., 2006). Interestingly, NOX4 not only contributes to TGFβ-mediated apoptosis, but also to death ligand (such as FasL or TNFα/actinomycin D)-induced hepatocyte apoptosis (Jiang et al., 2012). Hepatocyte-specific deletion of NOX4 reduced oxidative stress, lipid peroxidation and liver fibrosis in mice (Bettaieb et al., 2015). NOX4 was suggested to reduce the activity of the phosphatase PP1C, leading to prolonged activation of key stress signaling PKR/PERK pathway (Bettaieb et al., 2015). Therefore, NOX4 promotes myofibroblasts activation and hepatic fibrosis through at least two distinct mechanisms: (1) directly facilitating TGFβ-induced HSC activation and production of profibrogenic targets, (2) indirectly promoting TGFβ or death ligand-induced hepatocytes apoptosis, which contributes to the production of cytokines, chemokines, and microparticles that leads to HSC activation (Aoyama et al., 2012; Jiang et al., 2012).

Different from other NOX family proteins, the activity of NOX4 mainly depends on its expression levels, and not on agonist-induced assembly of a complex (Serrander et al., 2007). NOX4 predominantly mediates H_2_O_2_ production instead of superoxide (Martyn et al., 2006; Serrander et al., 2007). Potentially, H_2_O_2_ generated by NOX4 may contribute to the activation of certain protein tyrosine kinases that play crucial roles in TGFβ downstream signaling pathways (Bae et al., 2011). Hepatocytes and sinusoidal endothelial cells also express all of the components for NOX1 and NOX2 H (Jiang and Török, 2014). Owever, the mechanisms underlying NOX1/2 enzyme activation in these cells and their roles in regulating fibrosis and myofibroblasts activation remain largely unknown.

## Targeting hepatic fibrosis by inhibiting NOXs

Fibrosis is an intrinsic wound healing response that helps to maintain organ integrity upon severe tissue damage. However, fibrosis may also become problematic when persistent injury and sustained inflammation occurs. Unresolved liver fibrosis leads to accumulation of excessive ECM proteins and scarring, which eventually progresses to cirrhosis and HCC. As the key cells that produce fibrotic ECM and other fibrogenic components, hepatic myofibroblasts, and their products are considered primary targets for antifibrotic therapies (Schuppan and Kim, 2013; Tsochatzis et al., 2014). However, there is still no FDA-approved drug for the treatment of liver fibrosis. Accumulating evidence have suggested the critical pathogenic effects of oxidative stress in the development of liver fibrosis, therapies that target ROS using antioxidants have therefore been applied in pre-clinical models of liver diseases. For example, a natural antioxidant Pyrroloquinoline-quinone (PQQ) found in human foods, suppresses oxidative stress, and liver fibrogenesis in mice with attenuated liver damage, hepatic inflammation and activation of HSCs (Jia et al., 2015). Similarly, Silybin, an extract of silymarin with antioxidant and anti-inflammatory properties, has been shown to be hepatoprotective in rat livers with secondary biliary cirrhosis (Serviddio et al., 2014). Additionally, a recent study suggested that blocking chloride channels prevented the increase of intracellular superoxide anion radicals, leading to attenuated activation of HSCs (den Hartog et al., 2014). However, it should also be noted that several antioxidants have failed in clinical trials to demonstrate their efficacy in antifibrotic response, such as polyenylphosphatidylcholine in alcoholic liver disease (Lieber et al., 2003), and Ursodeoxycholic acid (UDCA) and vitamin E in NASH (Lindor et al., 2004; Sanyal et al., 2010).

Given the vital role of NOX and NOX-derived ROS in hepatic fibrogenesis, the use of novel pharmacological NOX inhibitors to treat patients with chronic liver disease is being considered as the most promising antifibrotic therapeutics. However, historical NOX inhibitors, such as apocynin and diphenylene iodonium (DPI), do not specifically target NOX-derived ROS and are not isoform specialized. Until recently, GenKyoTex (Geneva, Switzerland) has developed a first-in-class small molecule NOX1/NOX4 dual inhibitor (GKT137831), with little affinity for Nox2 isoform (Laleu et al., 2010). Inhibition of NOX1/NOX4 using GKT137831 attenuated CCl4 or BDL-induced ROS production and hepatic fibrosis in mice (Aoyama et al., 2012; Jiang et al., 2012; Lan et al., 2015). Mechanistically, GKT137831 suppressed profibrotic gene expression and ROS production in HSCs (Aoyama et al., 2012; Lan et al., 2015), and also decreased hepatocyte apoptosis (Jiang et al., 2012). GenKyoTex is finalizing Phase II clinical study, and GKT137831 displayed an excellent safety profile and statistically significant reduction in both liver enzyme and inflammatory marker levels. Together with results from pre-clinical animal models of various fibrotic disorders, NOX inhibition shows strong potential as an effective treatment for hepatic fibrosis. However, chronic liver diseases of different etiologies may require specific and/or combination antifibrotic treatment approaches, based on the fact that the crosstalk between different cell types is critical for myofibroblasts activation. Future, studies on the components and functions of specific NOX isoforms in specific cell types and specific liver diseases will provide deeper insights for designing more specific and potent NOX inhibitors for the treatment of hepatic fibrosis.

## Conclusions

Oxidative stress and inflammation are considered as the main cause of chronic liver diseases. Multiple lines of evidence indicate that NOX-generated ROS plays a pivotal role in the pathogenesis of liver fibrosis. A number of NOX isoforms, including NOX1, NOX2, and NOX4 are involved in the initiation of myofibroblasts activation and progression hepatic fibrosis. However, the intracellular pathways and molecular mechanisms involved in the role of NOX isoforms in specific cell types remain largely unknown. Targeting specific NOX isoforms with specific inhibitors, such as NOX1 and/or NOX4 to prevent HSC activation and protect hepatocyte injury may be promising to treat liver fibrosis, although future work is needed to fully confirm the clinical safety of these compounds. Moreover, the knowledge of molecular pathways involved in NOX-mediated myofibroblasts activation and fibrogenesis can provide new insights for developing novel anti-fibrotic treatments.

## Author contributions

SL wrote the manuscript, TK and DB revised the manuscript.

## Funding

NIH grants: (1) R01 DK099205; (2) P50 AA011999; (3) P42 ES010337.

### Conflict of interest statement

The authors declare that the research was conducted in the absence of any commercial or financial relationships that could be construed as a potential conflict of interest.
